# What can we do to enhance cognitive care in Parkinson's disease?

**DOI:** 10.1002/dad2.70276

**Published:** 2026-02-22

**Authors:** Dana Pourzinal, Deborah Brooks, John D. O'Sullivan, Sharon L. Naismith, Nadeeka N. Dissanayaka

**Affiliations:** ^1^ UQ Centre for Clinical Research The University of Queensland Herston Queensland Australia; ^2^ Department of Neurology Royal Brisbane & Women's Hospital Herston Queensland Australia; ^3^ Brain and Mind Centre The University of Sydney Sydney New South Wales Australia; ^4^ School of Psychology The University of Queensland Saint Lucia Queensland Australia

**Keywords:** cognitive impairment, dementia, guidelines, Parkinson's disease

## Abstract

For people living with Parkinson's disease (PD), cognitive impairment is a salient concern. The cumulative risk of mild cognitive impairment and dementia increases as the disease progresses, which contributes to loss of functional independence and increased institutionalization at late stages of PD. Yet, due to scarcity of time and resources, cognition is often an overlooked topic in busy neurology clinics. There are few opportunities for cognitive evaluation during clinical consults, and where it does occur, assessment methods are varied and unstandardized. Diagnostic and post‐diagnostic care pathways for cognitive decline vary across clinical services, leaving people with PD falling through the resonant cracks of our healthcare systems. This perspective article highlights current gaps and inconsistencies in the diagnosis, evaluation, and management of cognitive disorders in PD. In doing so, we explore ways in which some of these issues may be rectified through best practice guidelines and critically analyze those that are more difficult to address.

## Highlights

- Guidelines for cognitive disorders in PD are outdated.
- A literature search revealed key issues impacting diagnostic certainty and quality of care.
- There is a critical need for updated guidelines and standardized recommendations.
- A shift is occurring in persistent nihilistic attitudes and stronger emphasis on cognitive care.

## 1 INTRODUCTION

*“I can cope with physical changes, challenges, and limitations. But what if my intellect evaporates? That really concerns me, and I do not currently have any way to know or measure my likely pathway.”* −Stuart R (person living with young‐onset Parkinson's disease)

Updated guidelines for the diagnosis and evaluation of cognitive disorders in Parkinson's disease (PD) are long overdue. Cognitive decline is one of the highest rated concerns of people living with PD, which is unsurprising given its direct association with increased medical expenses and institutionalization.^1^, ^2^ Despite this, it has been just over a decade since the publication of *Movement Disorder Society* (MDS) criteria for mild cognitive impairment in PD (PD‐MCI) in 2012^3^ and almost two decades since publication of equivalent guidelines for PD dementia (PDD) in 2007.^4^ Since then, myriad studies have advanced our understanding of cognitive disorders in PD.^5^, ^6^, ^7^ Given that early and accurate detection of cognitive disorders is critical for the provision of care, we outline the urgent need for the development and implementation of comprehensive guidelines for diagnosis, evaluation, and management of cognitive disorders in PD. We highlight recent discoveries from the innovative Australian PDCogniCare framework to advance clinical evaluation and post‐diagnostic care for cognitive impairment and dementia in PD, closing the substantial gap between research and clinical practice.

## 2 SUBSTANTIAL GAPS IN CURRENT GUIDELINES

Our recent systematic review of recommendations for the diagnosis, evaluation, and post‐diagnostic care of cognitive disorders in PD revealed substantial gaps and inconsistencies.^8^ Here, we summarize our findings and provide critical suggestions for future work in this field.

### 2.1 Diagnosis

The most widely used criteria for diagnosing cognitive disorders in PD are the MDS criteria for PD‐MCI and PDD^3^, ^4^ and the Diagnostic Statistical Manual 5th Edition Text Revision (DSM‐5‐TR) criteria for mild and major neurocognitive disorder due to PD.^9^ In brief, the diagnosis of PD‐MCI or PDD requires PD to be clinically confirmed, cognitive decline to be objectively observed, and the onset of cognitive symptoms to occur at least 1 year after the onset of motor symptoms.^10^ Additionally, the DSM‐5‐TR diagnostic criteria for neurocognitive disorder due to PD necessitates insidious onset and gradual progression of impairment. The impairment must also not be attributable to another medical condition or better explained by another mental disorder.

We revealed significant gaps in the present approach to a diagnosis of cognitive disorders in PD, requiring further attention. First, the current diagnostic criteria do not clarify the role of biomarkers in the diagnostic process. Despite compounding evidence for biomarkers, there still remains the need for its clinical utility as diagnostic and prognostic markers for cognitive decline in PD. MDS guidelines for PD‐MCI advise against their use, while DSM‐5‐TR criteria make no reference at all; however, recent literature has supported specific biomarkers to improve diagnostic certainty of cognitive disorders in PD.^11^, ^12^, ^13^ Second, while cognitive subtyping can be useful for research purposes,^14^ it is unclear how to operationalize subtypes in a clinical setting. Providing clear criteria for cognitive subtyping in PD may be useful for the design and delivery of personalized interventions, for example by tailoring computerized cognitive training or strategy‐based training to specific cognitive symptoms.^15^ Finally, there is a distinct lack of guidance on how the diagnosis of cognitive disorders should be managed, for example, how much detail should be shared with the patient at the time of diagnosis, including disclosure of neuropsychological test results, and how patients with subjective, but not objective, cognitive decline should be characterized and managed. To address these gaps, the PDCogniCare framework conducted an inquiry into clinical pathways of cognitive evaluation in PD and identified areas where the integration of technology could reduce the complexity of current processes.^16^

### 2.2 Evaluation

MDS guidelines for PD‐MCI and PDD recommend cognitive evaluations at level 1, with brief measures of global cognitive function, or level 2, with a comprehensive cognitive test battery. PDD criteria suggest level 1 be used for dementia diagnosis, with level 2 recommended for qualitative exploration of impairments. PD‐MCI criteria allow diagnosis at either level as comprehensive testing is not always practical or available, while acknowledging reduced diagnostic certainty for level 1 compared to level 2 criteria. For this reason, level 2 PD‐MCI criteria demand administration of two tests across five cognitive domains (memory, executive function, language, visuospatial processing, and attention/working memory) and incorporate clinical thresholds to identify impairment, while level 2 PDD criteria do not. The clinical thresholds of the level 2 PD‐MCI criteria comprise either a score one to two standard deviations below demographically appropriate norms, a significant decline from previous performance on the test, or a significant decline from estimated premorbid levels. Impairment on two out of the 10 tests indicates PD‐MCI. Conversely, a diagnosis of PDD (or PD‐MCI at level 1) following MDS criteria only requires impairment on a measure of global cognitive function. The MDS criteria also make recommendations for measures to use in PD, including specific cognitive tests and measures of instrumental activities of daily living, given that PDD is primarily distinguished from PD‐MCI by the presence of functional impairment.

While these recommendations are aimed at standardizing cognitive evaluations in PD, their broad suggestions of clinical thresholds and measures also introduce methodological variability. This variability is a key contributor to the observed heterogeneity in cognitive function in PD, both in research and clinical settings. Understandably, guidelines err on the side of caution when recommending cognitive measures and thresholds due to differences in access to resources across clinics. However, heterogeneity in the methods used to assess cognition in PD influences its diagnostic and prognostic utility.^5^, ^6^, ^7^ Given that diagnostic accuracy for PD‐MCI and PDD improves with neuropsychological assessment,^17^ particularly when compared to diagnoses made by clinical judgment,^18^ homogeneity in methods of cognitive evaluation is critical to early identification of cognitive disorders in PD. A recent review by the PDCogniCare team explores this issue in depth and works toward developing a harmonized neuropsychological toolkit sensitive to cognitive decline in PD with specific considerations for administration in PD populations.^19^

Another significant gap we have identified is the lack of practical information, such as whether a neuropsychologist, neurologist, or specialized nurse should perform cognitive testing and discuss test results with patients, who to target with cognitive testing, and how frequently follow‐up testing should occur, particularly for those at risk of cognitive decline. Guidance on the administration of in‐home tele‐neuropsychology in PD for routine cognitive assessments is also lacking, which is relevant not only for those in rural areas without access to neuropsychology services but also for the many people with PD who are unable to access in‐person healthcare services due to mobility issues.^20^ While tele‐neuropsychology literature for presurgical workups in PD is established,^21^, ^22^ evidence for the diagnostic validity of tele‐neuropsychology in PD is only emerging^23^ and yet to be translated into practical guidelines for routine assessments. Additionally, while MDS guidelines acknowledge that impairment in activities of daily living due to cognitive impairment would be best assessed distinctly from those due to motor impairment, only recently have novel methods for actually achieving this been proposed on the basis that deficits within specific cognitive domains are linked to increased functional impairment in PD.^24^

### 2.3 Post‐diagnostic care

In terms of treatment options, pharmacological treatments for PDD are well characterized, with some evidence for acetylcholinesterase inhibitors, memantine, rasagiline, and atomoxetine.^2^ Conversely, recommendations for non‐pharmacological treatment options are underdeveloped. In particular, cognitive rehabilitation is generally overlooked despite growing evidence for computerized and strategy‐based cognitive training in PD.^25^, ^26^ These interventions have the potential for wide‐reaching impacts on quality of life, with evidence for their positive effect on motor function and activities of daily living.^27^, ^28^ Furthermore, care considerations are extremely limited, with most recommendations being derived from the National Institute for Health and Care Excellence (NICE) dementia guidelines. While evidence‐based recommendations for determining fitness to drive were well defined,^29^ referral pathways for people with cognitive disorders in PD were unclear, with only a small number of reviewed articles vaguely acknowledging the role of allied health professionals such as occupational therapists, psychologists, or movement disorder nurses in the management of cognitive impairment in PD. Importantly, discussions of advanced care directives are mostly referred to in the context of a PDD diagnosis and lack specific reference to medically assisted dying. This is likely due to the complexity of ethical concerns associated with these topics (e.g., consent, autonomy, duty of care), coupled with the fact that while assisted dying is available in Australia, it is not a universally accepted practice and, thus, not available in all countries. However, with current literature suggesting that such processes are a priority for people with MCI,^30^ clinicians would benefit from guidance on how to approach these sensitive topics. Other issues specific to people with cognitive disorders in PD such as managing polypharmacy, sleep disturbance, and mental ill health are not sufficiently addressed among guidelines, suggesting a misalignment between the literature and current demands in clinical practice.

## 3 ADDRESSING GAPS WITH REVISED GUIDELINES

In light of the above inquiry, there is a clear need for revised guidelines for the diagnosis, evaluation, and management of cognitive disorders in PD. Likewise, extensive discussion with our consumer and community involvement group, people living with PD, and their clinicians have revealed a strong sentiment for greater transparency and standardization of cognitive evaluations and post‐diagnostic processes, which was also supported by evidence from the United Kingdom.^31^ The PDCogniCare framework has developed guidelines informed by input from consumer/community experience, clinicians and researchers to improve diagnostic accuracy, patient flow, and post‐diagnostic care in clinical services for PD. Consensus in the development of evidence‐based recommendations was achieved via a two‐round modified Delphi procedure with 29 Australian clinician and research experts in PD^32^ and a national survey of 81 people with lived experience of PD,^33^ informing easily adoptable best practice guidelines that are accessible to all PD clinicians. In doing so, we strive to promote awareness of cognitive disorders in PD, with the ultimate goal of improving quality of care and quality of life for people with PD.

Naturally, skepticism toward increased cognitive testing in PD arises from the limited effective treatment options. Only some acetylcholinesterase inhibitors, generally prescribed for Alzheimer's disease (AD), have been shown to be efficacious in PDD but not PD‐MCI.^34^, ^35^ Why risk distressing patients with a diagnosis for which we have limited treatments? We suggest the skeptics consider instead the impact that accurate early detection of cognitive impairment will have on other aspects of patient care. For example, identifying people with PD at risk of cognitive decline in a timely manner will enable more effective (1) enrollment of patients in preventive clinical trials, (2) advanced care planning prior to impaired capacity, (3) allocation of healthcare services to people who will derive the most benefit, and (4) policy decision‐making and advocacy, particularly regarding contentious issues such as voluntary assisted dying for people living with PDD. Early diagnosis of cognitive disorders empowers individuals to make informed decisions while they have the capacity to do so, promoting autonomy, dignity, and person‐centered care. Failure to identify cognitive impairment in PD hinders early treatment, rehabilitation potential, psychosocial support and education, and future planning for the person and their careers. The PDCogniCare team recognizes the potential for improved guidelines for cognitive disorders to reduce the uncertainty in diagnosis and post‐diagnostic pathways and meaningfully enhance patient‐centered care and shared decision‐making in PD.

## 4 WHERE TO NEXT?

As the concept of cognitive care in PD evolves, newly developing areas of clinical practice and research will inform future iterations of the guidelines. For instance, recent evidence suggests that greater integration of palliative care, particularly prior to late stages of cognitive decline where communication and capacity are compromised, improves end‐of‐life care and quality of life for people with PD.^36^ The integration of dementia risk stratification, particularly using genetic markers such as GBA and APOE ε4, in clinical decision‐making for people with PD is also an emerging practice, with recent recommendations made to guide symptomatic therapy.^37^ Similarly, the strong association between AD co‐pathology and increased dementia risk in PD^38^, ^39^ has signaled the potential for clinical use of blood‐based AD biomarkers to detect early cognitive decline in PD.^40^

Furthermore, while PDCogniCare focuses on cognitive disorders in PD to align with current models of care in movement disorders clinics, this clinical approach is expected to change as the single entity theory of neuronal α‐synuclein disease develops.^41^ In particular, dementia with Lewy bodies (DLB) and PD share a common α‐synuclein pathology and exhibit substantial overlap in cognitive, neuropsychiatric, and functional features. The diagnostic criteria and neuropsychological profiles of MCI and dementia in PD and DLB overlap considerably, complicating nosological distinctions and clinical classification. The gaps in cognitive care and novel consensus recommendations highlighted here are thus likely to extend beyond PD alone, with the potential to promote stronger management of cognitive symptoms across the spectrum of neuronal α‐synucleinopathies.

Recent advancements to enhance early diagnosis, prognosis, and treatment of cognitive disorders also provide insight into the not‐so‐distant future of clinical practice for people with PD. Emerging biomarker literature validating novel cerebrospinal fluid, imaging, and plasma markers of cognitive impairment demonstrate utility for identifying people with PD at risk of dementia.^13^ Artificial intelligence has also shown profound potential to revolutionize the diagnosis and management of cognitive disorders, especially through the integration of multimodal data to produce person‐centered treatment interventions and precision medicine.^42^, ^43^ Finally, innovations in disease‐modifying therapies such as monoclonal antibodies in the treatment of AD and gene therapy targeting PD motor symptoms instill hope for the future of interventions for cognitive disorders in PD.^44^, ^45^ Clinical guidelines should endeavor to keep up with these rapid technological advancements to maintain currency of evidence‐based practices and high quality of care for people living with PD.

## 5 CONCLUSION

Our work strives to change the nature of how we view the cognitive symptoms of PD. By creating revised guidelines for assessing and managing cognitive disorders in PD, we aim to draw greater attention to a problem that has long been a priority of the patient and make it a priority in the clinic. Given that there are typically few opportunities for thorough cognitive evaluation in current PD clinical care, the PDCogniCare project aims to address the specific needs of people living with PD and cognitive impairment by encouraging more purposeful and targeted cognitive assessment and post‐diagnostic care options. While we acknowledge the current limited treatment options, our work builds on previous guidelines to improve early detection of cognitive impairment in PD to advance person‐centered care and preventive interventions. In doing so, we champion a cultural shift around diagnosing and characterizing MCI and dementia in PD, where the question is no longer “What is the point?” but rather “What can we do?”

## CONFLICT OF INTEREST STATEMENT

Dana Pourzinal has received funding from the Medical Research Future Fund (MRFF) Dementia, Ageing and Aged Care (DAAC) grant (PDCogniCare), honoraria for student supervision, and travel grant funding from the Movement Disorder Society and has shares in BrainChip. Deborah Brooks has received funding from the MRFF DAAC grant (PDCogniCare). John O'Sullivan is an advisory board member for AbbVie and STADA. Alexander Lehn receives payment or honoraria from AbbVie and STADA, is an advisory board member for AbbVie, has received travel grant funding from the Movement Disorder Society, and is a board member for Parkinson's Queensland. Sharon Naismith does consultancy work for Eisai and Roche, is on the advisory board for Eisai, is employed by the University of Sydney, and receives grant funding from the National Health and Medical Research Council (NHMRC), Alzheimer's International, Alzheimer's Drug Discovery Foundation, and Medical Research Future Fund. Nadeeka Dissanayaka receives consulting fees from the NHMRC, has a leadership role in the Australian Dementia Network Early to Mid‐Career Researcher Accelerator Group, receives grant funding from NHMRC MRFF (PDCogniCare), a NHMRC Boosting Dementia Research Leadership Fellowship, UQ Amplify Research Fellowship, auDA Community Grant, Dementia Australia Research Foundation, and The Prince Charles Hospital Foundation and has received philanthropic donations for research. Author disclosures are available in the Supporting Information.

## Supporting information



Supporting information

## Data Availability

The authors have nothing to report.
